# Selective Estrogen Receptor Modulator-Associated Nonalcoholic Fatty Liver Disease Improved Survival in Patients With Breast Cancer: A Retrospective Cohort Analysis

**DOI:** 10.1097/MD.0000000000001718

**Published:** 2015-10-09

**Authors:** Qiufan Zheng, Fei Xu, Man Nie, Wen Xia, Tao Qin, Ge Qin, Xin An, Cong Xue, Roujun Peng, Zhongyu Yuan, Yanxia Shi, Shusen Wang

**Affiliations:** From the Department of Medical Oncology, Sun Yat-sen University Cancer Center; State Key Laboratory of Oncology in South China; Collaborative Innovation Center for Cancer Medicine, Guangzhou, Guangdong, PR China.

## Abstract

Selective estrogen receptor modulator (SERM)-associated nonalcoholic fatty liver disease (NAFLD) might be related to treatment efficacy in patients with breast cancer because of circulating estrogen antagonism.

The aim of the study was to investigate the relationship between NAFLD and survival outcomes in patients with breast cancer who were treated with tamoxifen or toremifene.

This single-center, retrospective, cohort study included 785 eligible patients who received tamoxifen or toremifene, after curative resection for breast cancer, at the Sun Yat-sen University Cancer Center between January 2005 and December 2009. Data were extracted from patient medical records. All patients underwent abdominal ultrasonography, at least once, at baseline and at the annual follow-up. Patients who were diagnosed with NAFLD on ultrasonography were classified into the NAFLD or the non-NAFLD arm at the 3-year follow-up visit. Univariate and multivariate Cox regression analyses were conducted to evaluate any associations between NAFLD and disease-free survival (DFS) or overall survival (OS).

One hundred fifty-eight patients were diagnosed with NAFLD. Patients who developed NAFLD had better DFS and OS compared with those who did not. Univariate analyses revealed that the 5-year DFS rates were 91.56% and 85.01% for the NAFLD and non-NAFLD arms, respectively (hazard ratio [HR], 0.59; 95% confidence interval [CI], 0.37–0.96; log-rank *P* = 0.032). The 5-year OS rates were 96.64% and 93.31% for the NAFLD and non-NAFLD arms, respectively (HR, 0.39; 95% CI, 0.16–0.99; log-rank *P* = 0.039). Multivariate analysis revealed that NAFLD was an independent prognostic factor for DFS, improving the DFS rate by 41% compared with that in the non-NAFLD arm (HR, 0.59; 95% CI, 0.36–0.96; *P* = 0.033).

SERM-associated NAFLD was independently associated with improved DFS and might be useful for predicting treatment responses in breast cancer patients treated with SERMs.

## INTRODUCTION

In China, breast cancer is the most prevalent cancer among urban women and the fourth leading cancer in rural areas.^1^ For Chinese women, the mean age at diagnosis is 45 to 55 years, which is significantly less in comparison with that in Western women.^1^ Epidemiological studies show that approximately 60% of Chinese women are premenopausal when diagnosed with breast cancer.^2,3^ Consequently, in China, selective estrogen receptor modulators (SERMs) are standard endocrine therapeutic agents.

Tamoxifen and toremifene are SERMs with proven efficacy in patients with hormone receptor-positive breast cancer. A multicenter randomized trial showed that both drugs had equal efficacy and similar side effect profiles in the adjuvant setting for node-positive postmenopausal women with early breast cancer.^4^ Similarly, the International Breast Cancer Study Group Trials 12-93 and 14-93 demonstrated that both drugs had consistent outcomes, toxicities, and effects on patient quality of life.^5^

Even though significant outcome improvements have been gained using endocrine therapy, not all patients benefit from SERMs. Clinically, long-term administration of tamoxifen and toremifene caused frequent side effects, including climacteric symptoms, venous thromboembolism, and lipid dysfunction such as nonalcoholic fatty liver disease (NAFLD).^6–9^ It has been reported that approximately 40% of patients undergoing tamoxifen treatment developed NAFLD within 2 years.^6,7,10^ The pathogenesis of SERM-associated NAFLD has not been clearly defined, but is under active investigation. Inhibition of fatty acid oxidation and promotion of triacylglycerol biosynthesis have been suggested as putative mechanisms.^11–13^ In vitro studies have demonstrated that tamoxifen can induce hepatocyte steatosis and increase hepatocyte triglyceride accumulation by modulating the expression of genes involved in the triglyceride homeostasis pathway.^14,15^ Furthermore, estrogen can regulate fatty acid oxidation.^16^

SERMs bind to and block the estrogen receptor (ER), acting as potent inhibitors of estrogen signaling, which could weaken fatty acid oxidation and result in NAFLD development. Consequently, whether there is an association with the antitumor potency of SERMs is unknown. We performed a retrospective study to evaluate the hypothesis that development of NAFLD in SERM-treated patients with breast cancer improved SERM antitumor efficacy.

## PATIENTS AND METHODS

### Patients

**FIGURE 1:**
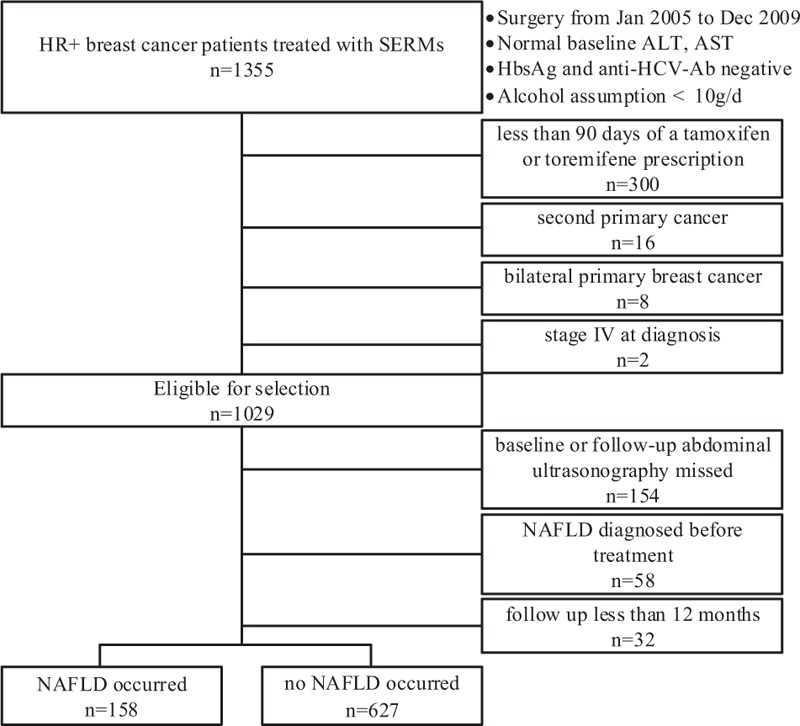
Flow diagram showing the exclusion criteria of the study. ALT = alanine aminotransferase, anti-HCV-Ab = anti-HCV antibody, AST = aspartic aminotransferase, NAFLD = nonalcoholic fatty liver disease, SERMs = selective estrogen receptor modulators.

After being granted approval by Institutional Review Board of Sun Yat-sen University Cancer Center, Guangzhou, China, we conducted a retrospective study by searching electronic medical records to identify patients with primary invasive breast cancer. We identified 1355 patients who had undergone curative surgery between January 2005 and December 2009. All 1355 patients had immunohistochemically confirmed ER/progesterone receptor (PgR)-positive breast cancer, normal baseline liver function, and complete covariate information in the medical records. In addition, all patients were hepatitis B surface antigen-negative and antihepatitis C virus antibody-negative and consumed <10 g of alcohol per day. Exclusion criteria are shown in Figure 1. None of the patients had NAFLD according to baseline ultrasonography. Finally, 785 eligible patients with breast cancer treated with tamoxifen or toremifene were included. Written informed consent was provided by all patients.

### Study Design

The clinical endpoints were disease-free survival (DFS) and overall survival (OS). DFS was defined as the time from the date of surgery until the date of the earliest documentation of disease recurrence, metastasis, or death from any cause, or until the end of follow-up. OS was defined as the time from the date of surgery until the date of death from any cause, or until the end of follow-up. Clinicopathological characteristics were evaluated, including age at diagnosis, body mass index (BMI) before SERM administration, tumor size, nodal status, histologic grade, tumor–node–metastasis (TNM) stage, lymphovascular invasion, ER expression, PgR expression, human epidermal growth factor receptor 2 (HER2) status, and treatment regimen (chemotherapy, radiotherapy, surgery, endocrine therapy). The duration of SERM use was calculated by subtracting the first SERM prescription date from the last day of coverage. BMI was defined as weight/height^2^ (kg/m^2^). Tumor staging was based on the seventh edition of the American Joint Committee on Cancer criteria. ER and PgR status were designated as binary variables according to a cutoff value of 10%. HER2-positivity was confirmed as immunohistochemical 3+ staining intensity or amplification detected by fluorescence in situ hybridization.

### NAFLD Assessment

All patients underwent abdominal ultrasonography at least once at baseline and once at the annual follow-up visit. Patients were asked to consume a fat-free meal on the evening before the test and to avoid eating for 4 to 8 hours before the test. A 3- to 5-MHz transducer and ACUSON Sequoia^TM^ 512 ultrasound machine (Siemens, Mountain View, CA) were used for upper abdominal and hepatic imaging. Ultrasonography was performed by experienced sonographers who were blinded to the study aims and data. The diagnosis of NAFLD was based on an increase in liver echogenicity, discrepancies between hepatic and renal echoes, and echo loss from the portal vein walls.^17^

### Statistical Analyses

Patients were classified into the NAFLD and non-NAFLD arms at the first 3-year follow-up visit. Mann–Whitney *U* and χ^2^ tests were used to compare continuous and categorical data, respectively. Multivariate logistic regression analysis was used to identify independent factors associated with the development of NAFLD. The Kaplan–Meier method was used to calculate the cumulative survival rate and to plot DFS and OS curves. The log-rank test was used to compare the differences in DFS and OS between the study arms. Univariate Cox regression analysis was used to identify prognostic factors associated with DFS and OS. The multivariate Cox proportional hazards model with a forward stepwise selection of significant univariate factors or probably important confounding variables was applied to determine which factors acted as independent prognosticators. All statistical analyses were conducted using SPSS 19.0 for Windows (SPSS Inc, Chicago, IL). A 2-tailed *P* value of ≤0.05 was considered statistically significant.

## RESULTS

### Patient Characteristics

The median follow-up was 76 months (range, 14–122 months). The cumulative 1, 2, and 3-year NAFLD development rates were 10.8%, 16.2%, and 20.1%, respectively. The NAFLD arm comprised 158 patients, and the non-NAFLD arm comprised 627 patients. Baseline patient characteristics for both arms are shown in Table 1 . The median age at diagnosis was 45 years in the NAFLD arm and 42 years in the non-NAFLD arm. There were no significant differences in tumor size, lymph node-positive number, histologic grade, TNM stage, ER or PgR expression, HER2 status, or treatment regimens between the arms. Patients in the NAFLD arm were significantly older and had high BMI compared with those in the non-NAFLD arm (both *P* < 0.001).

**TABLE 1:**
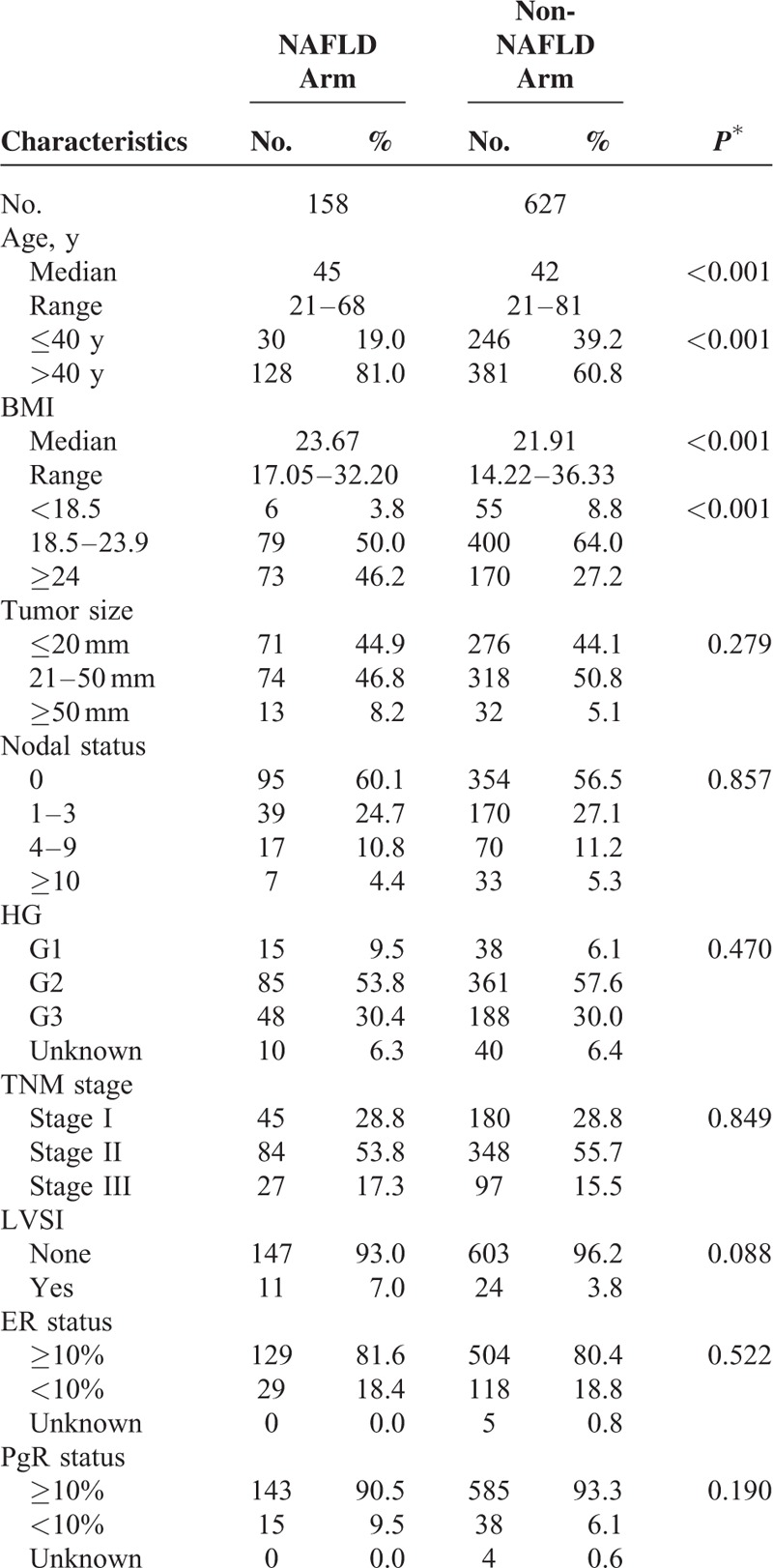
Baseline Characteristics Grouped by Nonalcoholic Fatty Liver Disease Developed or Not

**TABLE 1 (Continued):**
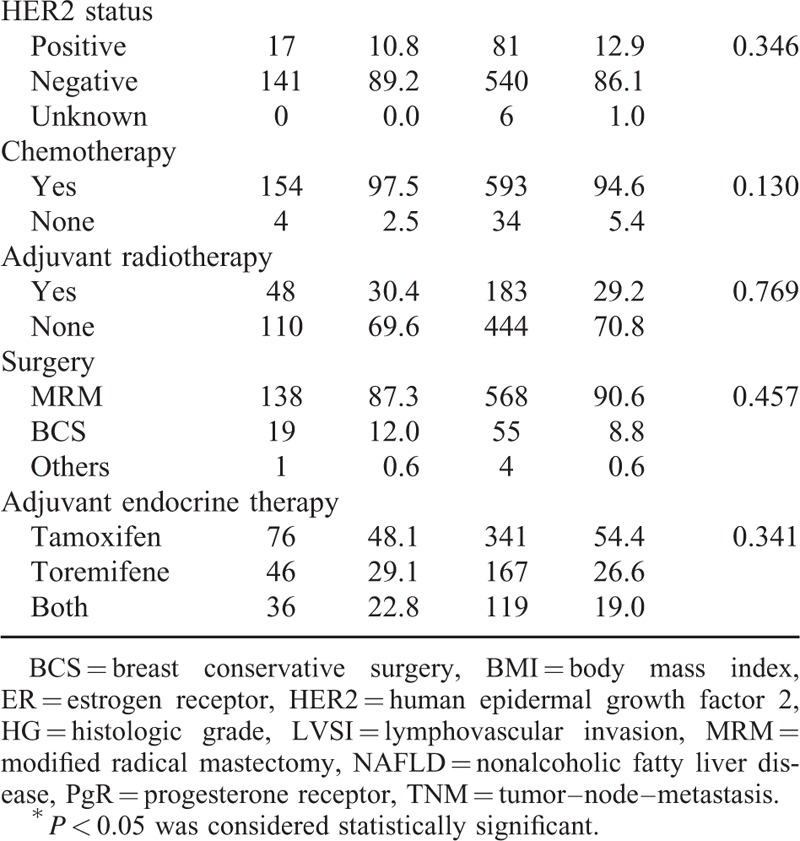
Baseline Characteristics Grouped by Nonalcoholic Fatty Liver Disease Developed or Not

In the NAFLD arm, of the 154 (97.5%) patients who underwent chemotherapy, 151 were administered anthracycline and/or taxane-based regimens. In the non-NAFLD arm, of the 593 patients who underwent chemotherapy, 577 were administered anthracycline and/or taxane-based regimens.

### Baseline Characteristics Associated With NAFLD Development

Multivariate logistic regression analysis of independent factors associated with NAFLD development is shown in Table 2. Age and BMI were positively associated with NAFLD development (age: odds ratio [OR], 1.04; 95% confidence interval [CI], 1.02–1.07, *P* = 0.001; BMI: OR, 1.11; 95% CI, 1.05–1.18; *P* = 0.001). In addition, chemotherapy administration (OR, 5.51; 95% CI, 1.52–20.00; *P* = 0.01) was significantly associated with NAFLD development.

**TABLE 2:**
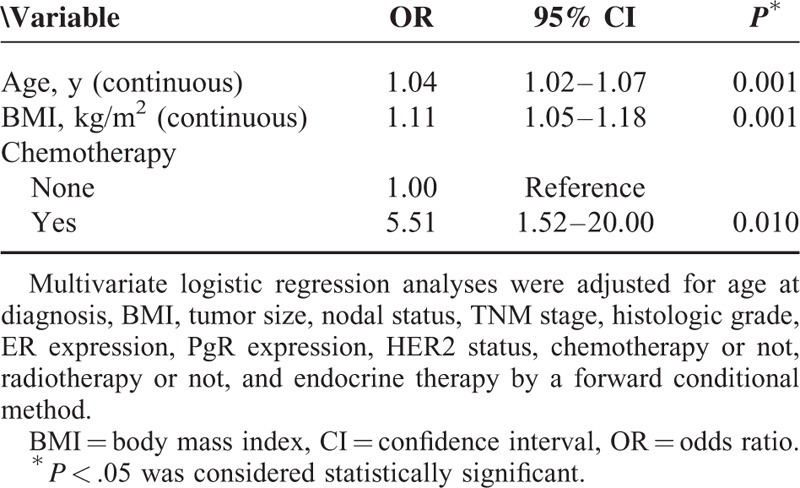
Multivariate Analysis of Factors Related to Nonalcoholic Fatty Liver Disease Developed

### The Effect of NAFLD Development on DFS

**FIGURE 2:**
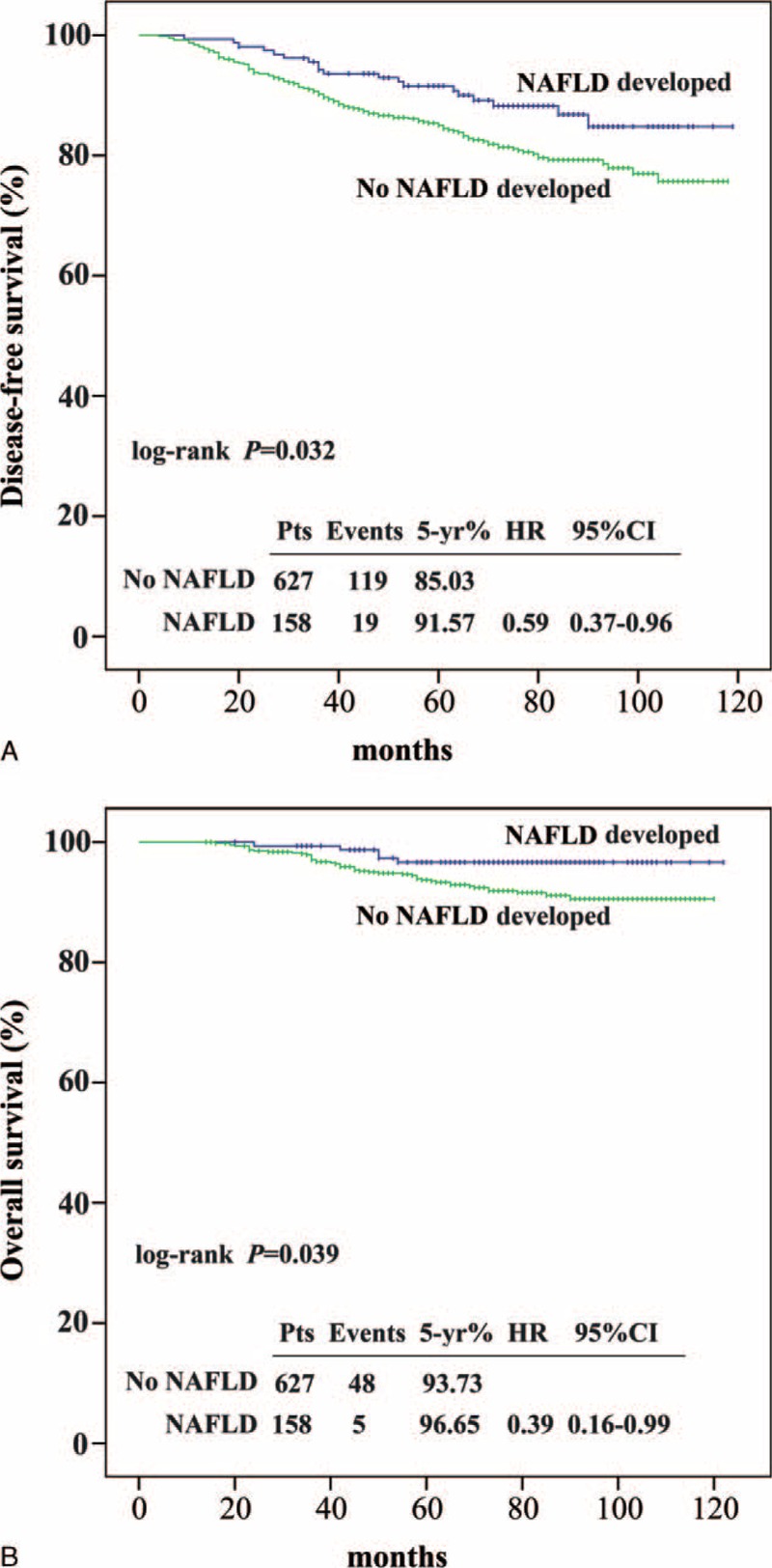
Kaplan–Meier curves for disease-free survival (A) and overall survival (B) according to the nonalcoholic fatty liver disease developed or not. Median follow-up was 76 months. CI = confidence interval, HR = hazard ratio, NAFLD = nonalcoholic fatty liver disease.

In the univariate analysis, NAFLD development, smaller tumor size, less lymph node metastasis, earlier TNM stage, lower histologic grade, high ER expression, HER2-negative status, no chemotherapy, and no radiotherapy were associated with improved DFS (Table 3). The 5-year DFS rates were 91.57% and 85.03% in the NAFLD and non-NAFLD arms, respectively (log-rank test: *P* = 0.032; univariate hazard ratio [HR], 0.59; 95% CI, 0.37–0.96) (Figure 2A). In the age- and BMI-adjusted multivariate analysis, NAFLD development was confirmed as an independent prognosticator for DFS. Patients in the NAFLD arm who underwent SERM treatment had a 41% reduction in disease risk (multivariate HR, 0.59; 95% CI, 0.36–0.96; *P* = 0.033) compared with patients in the non-NAFLD arm (Table 4).

**TABLE 3:**
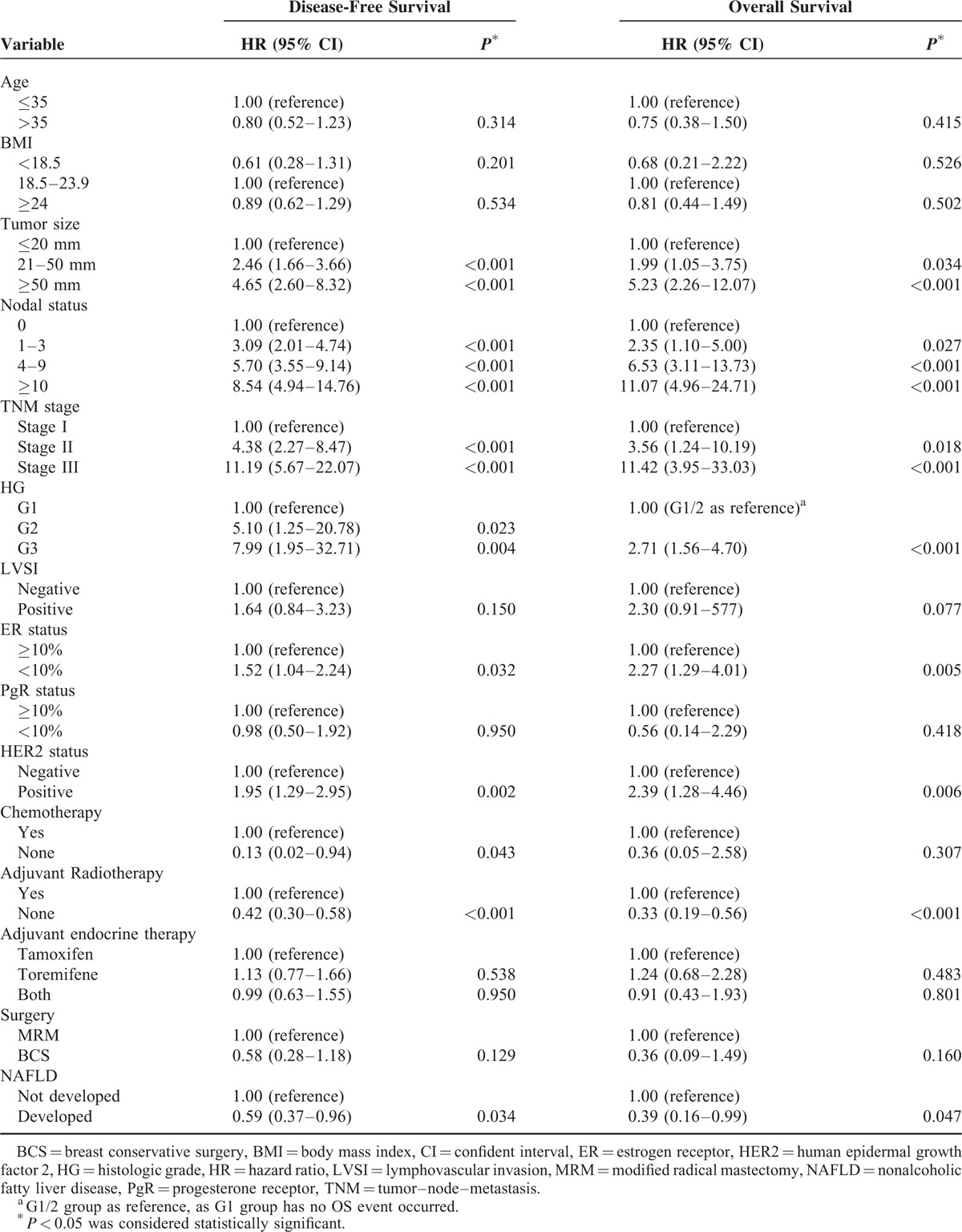
Univariate Analysis of Prognostic Factors Related to Disease-Free Survival and Overall Survival in Patients With Breast Cancer

**TABLE 4:**
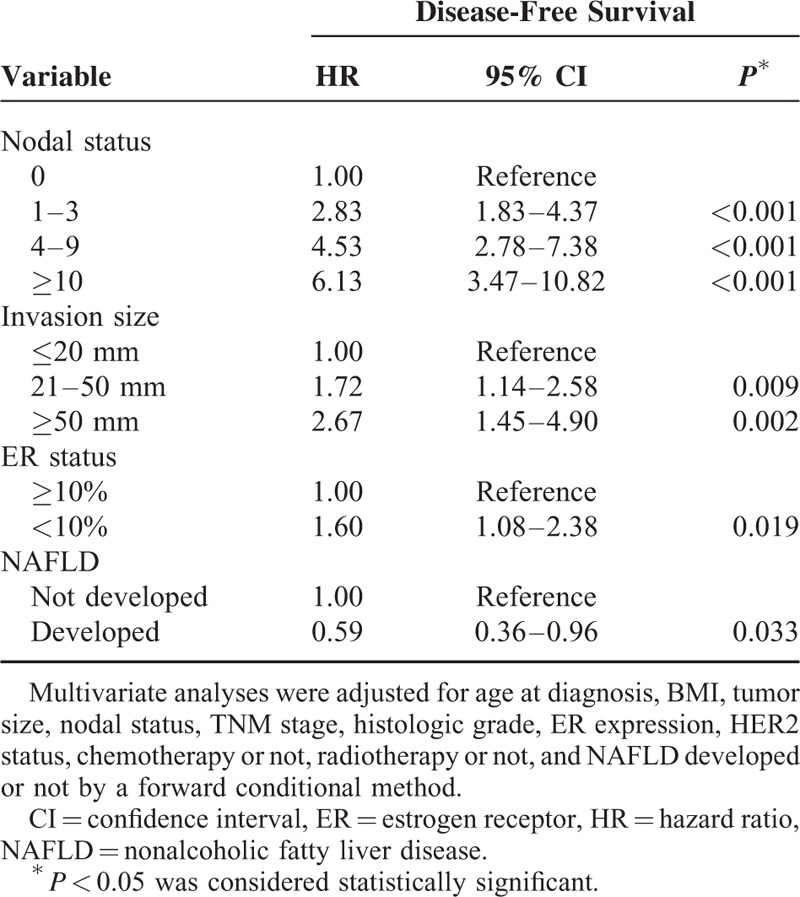
Multivariate Analysis of Prognostic Factors Related to Disease-Free Survival in Patients With Breast Cancer

### The Effect of NAFLD Development on OS

Table 3 and Figure 2B show the OS outcomes. The 5-year OS rates were 96.65% and 93.73% in the NAFLD and non-NAFLD arms, respectively (log-rank test *P* = 0.039). Univariate analysis revealed that patients in the NAFLD arm had longer OS compared with those in the non-NAFLD arm (HR, 0.39; 95% CI, 0.16–0.99; *P* = 0.047) (Table 3). However, after adjustment for other conventional prognostic factors, NAFLD was not an independent prognosticator for OS. Nodal status, ER expression, and histologic grade were independent prognosticators for OS (N1 vs N0, *P* = 0.018; N2 vs N0, *P* < 0.001; N3 vs N0, *P* < 0.001; ER ≥10% vs ER < 10%, *P* = 0.005; G3 vs G1/2, *P* = 0.017) (Table 5).

**TABLE 5:**
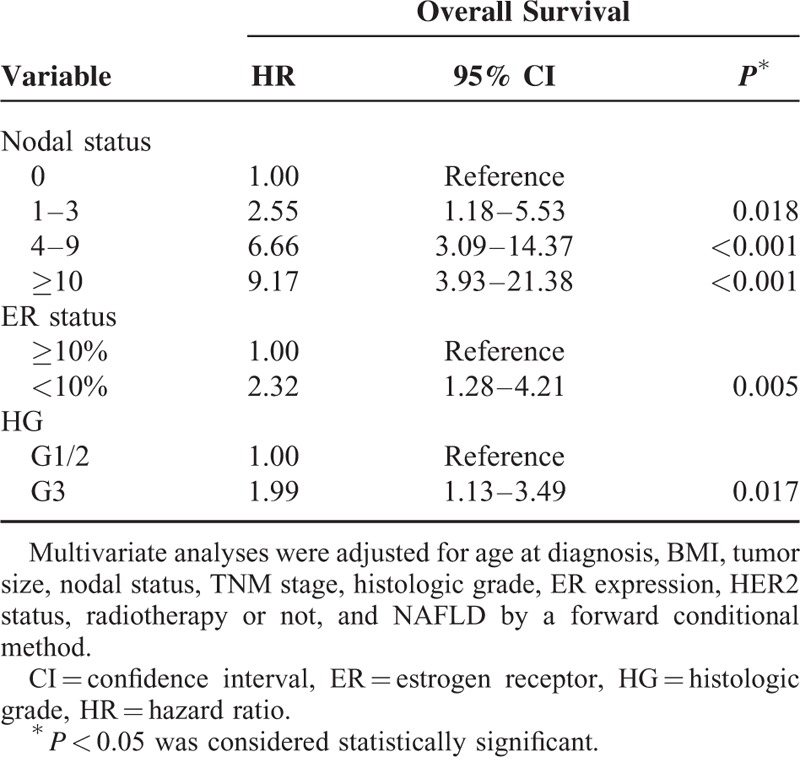
Multivariate Analysis of Prognostic Factors Related to Overall Survival in Patients With Breast Cancer

## DISCUSSION

In this retrospective study, significantly better DFS was observed in patients with early breast cancer who developed NAFLD in the first 3 years of SERM treatment compared with those who did not develop NAFLD. This is the first study, to our knowledge, to report that SERM-associated NAFLD plays a protective role in early breast cancer. In addition, nodal status, invasion size, and ER expression were independent prognosticators for DFS.

The diagnosis of drug-associated NAFLD is challenging; it remains a clinical diagnosis of exclusion because it lacks objective diagnostic tests. However, a reliable diagnosis can be made by a thorough analysis of patient history and clinical, laboratory, and imaging examinations. A prospective, randomized control study indicated that tamoxifen-treated patients had a 3-year cumulative NAFLD incidence of 32.6% compared with 9.6% for that in anastrozole-treated patients.^10^ Likewise, some retrospective studies have reported an NAFLD incidence rate of approximately 40% in tamoxifen-treated patients.^6,7^ Consequently, we have reason to hypothesize that NAFLD might be correlated with adjuvant endocrine therapy.

In our study, the cumulative 1, 2, and 3-year NAFLD development rates were lower compared with those reported in previous studies.^6,7,10^ This was most probably because when compared with other study cohorts, our patients were younger and included more number of premenopausal women. In addition, we used ultrasonography as a diagnostic instrument, rather than computed tomography that could lead to differential findings between studies. Moreover, some patients undergoing SERM treatment switched between tamoxifen and toremifene regimens, and it has been demonstrated that toremifene is less likely to induce NAFLD compared with tamoxifen.^18^ In Western countries, there is little evidence to suggest the use of toremifene in premenopausal women; however, in China, toremifene has been proposed as an alternative to tamoxifen in premenopausal women.

It is well known that some drug-related side effects can positively affect treatment efficacy. The International Tamoxifen Exemestane Adjuvant Multinational (TEAM) trial found that patients who reported treatment-related adverse events, such as vasomotor symptoms, musculoskeletal and joint symptoms, and vulvovaginal symptoms during the first year of treatment, had improved DFS and OS and fewer distant metastases compared with those who did not.^8^ In addition, patients who experienced mammographic breast density reduction during tamoxifen treatment also had better outcomes.^19–21^ In the present study, NAFLD, a drug-specific adverse event, improved DFS in SERM-treated patients with hormone receptor-positive breast cancer. This evidence could be useful for clinicians in encouraging patients with treatment-related adverse events to persist with long-term endocrine therapy. More importantly, those who do not experience treatment-related adverse events should undergo more frequent follow-up and attention. However, management of toxicity during SERM treatment should not be viewed lightly because NAFLD could progress to nonalcoholic steatohepatitis, or even irreversible liver cirrhosis.^22^ Diet modifications and treatment with pharmacologic agents have been used to manage drug-associated liver steatosis, but randomized control trials of their efficacy have not been conducted.^23^

NAFLD is a well-known risk factor for various malignancies.^24–26^ However, some studies have suggested a protective role for NAFLD in patients with malignancies.^27,28^ One demonstrated that the time to prostate cancer biochemical recurrence after radical prostatectomy was significantly delayed in patients with NAFLD.^27^ Another illustrated that colorectal cancer patients with NAFLD at baseline had higher OS rates compared with the non-NAFLD patients.^28^ In our study, although NAFLD was SERM-associated, it similarly improved DFS in NAFLD patients. The mechanisms involved may be analogous with those in other studies, and probably involved the insulin and insulin-like growth factor (IGF) axis, which are well recognized as promoters of cellular proliferation and antiapoptosis.^29^ The IGF-1 receptor (IGF-1R) and insulin receptors are functional in all breast cancer subtypes.^30–32^ IGF-1R signaling can mediate antiestrogen resistance via cross talk with ER signaling. In ER-positive MCF7 human breast cancer cells, overexpression of IGF-1R increased receptor tyrosine kinase activity in response to IGF-1 ligand stimulation, promoting resistance to tamoxifen and fulvestrant.^33^ NAFLD has been shown to be associated with decreased circulating IGF-1 levels,^34,35^ which might partially explain why NAFLD was associated with better breast cancer outcomes during SERMs treatment. Currently, the number of drugs developed to target the IGF-1 and insulin receptors has increased, and a series of phase 1 and 2 studies are underway. However, for advanced breast cancer, the preliminary findings from these studies have been controversial. Phase I studies showed that the IGF-1R inhibitors, ganitumab (AMG 479), AVE1642, R1507, and cixutumumab had promising activity against metastatic breast cancer,^36–39^ but a phase II study of ganitumab in combination with endocrine therapy did not improve the outcomes in patients with endocrine-resistant hormone-receptor-responsive metastatic breast cancer.^40^ These studies suggest that not all patients would benefit from IGF-1R inhibitor therapy; therefore, more studies, including biomarker analysis, are needed to validate the usefulness of IGF-1R inhibitors in breast cancer patients.

The present study had limitations. First, exact information on the menopausal status of all the patients was not available, yet the menopausal status was a probable confounding factor of NAFLD and was associated with the prognosis of early breast cancer. In addition, suppression of ovarian function was not routinely performed in high-risk and young patients. Second, we used ultrasonography to diagnose NAFLD, whereas the gold standard is a liver biopsy. We did not grade the severity of liver steatosis or classify the pattern of steatosis. Furthermore, we could not differentiate steatohepatitis from steatosis because this is not possible using ultrasonography; therefore, the severity-related effect of steatosis could not be evaluated. Third, because of the retrospective study design, there may be an element of selection bias. Furthermore, we could not control for confounding factors that might influence test results for serum triglycerides or liver enzyme levels, so they were not evaluated. Well-designed, prospective cohort studies are needed to validate our findings.

In conclusion, in patients with breast cancer, SERM-associated NAFLD was an independent prognosticator associated with improved DFS. In addition, knowing that SERM-associated NAFLD is protective could be useful for clinicians to persuading patients who experience side effects to continue with the treatment regimen. Conversely, it may also indicate that those who do not develop NAFLD should be followed more closely.
